# Gene expression alterations related to mania and psychosis in peripheral blood of patients with a first episode of psychosis

**DOI:** 10.1038/tp.2016.159

**Published:** 2016-10-04

**Authors:** E S Gouvea, V K Ota, C Noto, M L Santoro, L M Spindola, P N Moretti, C M Carvalho, G Xavier, A C Rios, J R Sato, M A F Hayashi, E Brietzke, A Gadelha, R A Bressan, Q Cordeiro, S I Belangero

**Affiliations:** 1 Department of Psychiatry, Universidade Federal de São Paulo, São Paulo, Brazil; 2 Department of Psychiatry, Irmandade da Santa Casa de Misericórdia de São Paulo, São Paulo, Brazil; 3 Genetics Division, Department of Morphology and Genetics, UNIFESP, São Paulo, Brazil; 4 LiNC—Interdisciplinary Laboratory of Clinical Neurosciences, UNIFESP, São Paulo, Brazil; 5 Center of Mathematics, Computation and Cognition, Universidade Federal do ABC, São Paulo, Brazil; 6 Department of Pharmacology, UNIFESP, São Paulo, Brazil; 7 Research Group in Behavioral and Molecular Neuroscience of Bipolar Disorder, UNIFESP, São Paulo, Brazil

## Abstract

Psychotic disorders affect ~3% of the general population and are among the most severe forms of mental diseases. In early stages of psychosis, clinical aspects may be difficult to distinguish from one another. Undifferentiated psychopathology at the first-episode of psychosis (FEP) highlights the need for biomarkers that can improve and refine differential diagnosis. We investigated gene expression differences between patients with FEP–schizophrenia spectrum (SCZ; *N*=53) or FEP–Mania (BD; *N*=16) and healthy controls (*N*=73). We also verified whether gene expression was correlated to severity of psychotic, manic, depressive symptoms and/or functional impairment. All participants were antipsychotic-naive. After the psychiatric interview, blood samples were collected and the expression of 12 psychotic-disorder-related genes was evaluated by quantitative PCR. *AKT1* and *DICER1* expression levels were higher in BD patients compared with that in SCZ patients and healthy controls, suggesting that expression of these genes is associated more specifically to manic features. Furthermore, *MBP* and *NDEL1* expression levels were higher in SCZ and BD patients than in healthy controls, indicating that these genes are psychosis related (independent of diagnosis). No correlation was found between gene expression and severity of symptoms or functional impairment. Our findings suggest that genes related to neurodevelopment are altered in psychotic disorders, and some might support the differential diagnosis between schizophrenia and bipolar disorder, with a potential impact on the treatment of these disorders.

## Introduction

Psychotic disorders, including schizophrenia, bipolar disorder and schizoaffective disorder, affect ~3% of the general population^1, 2^ and represent some of the most severe mental diseases. Characteristic symptoms include hallucinations, delusional beliefs, severe mood variations and cognitive impairment. However, during the early stages of psychosis, the clinical aspects may be difficult to distinguish from one another. The first-episode psychosis (FEP) is a critical period given that brain abnormalities and cognitive deficits are already present and progress faster, and more aggressively in the first years of the disorder,^3^ whereas the patients are not affected yet by factors related to disease progression, that is, duration of illness and exposure to antipsychotics.^4, 5^

More than a century has passed since Kraepelin first proposed the distinction between dementia praecox (schizophrenia) and manic-depressive insanity (bipolar disorder),^6^ but as both disorders may share the same psychotic symptoms, differentiating schizophrenia spectrum disorders from bipolar disorder is still a challenge. Therefore, different lines of research aim to identify biomarkers capable of distinguishing these disorders, including studies based on gene expression in peripheral tissues.^7^ On the other hand, some genes, including microRNAs, show a concordant expression and association for both schizophrenia and bipolar disorder in blood^8^ and also in brain tissues,^9, 10^ showing a possible common pathophysiological mechanism between these disorders, beyond the diagnostic boundaries. Moreover, previous studies revealed an effect of antipsychotics on gene expression.^11, 12, 13^ Therefore, assessing gene expression in early stages, such as FEP, is crucial, particularly before the administration of antipsychotics, but this is only feasible in peripheral tissues.

The majority of studies have focused on schizophrenia-spectrum psychosis, suggesting alterations in genes related to myelination, neurodevelopment and AKT pathway,^14^ although affective psychoses studies are under-represented in the literature, particularly early-stage affective psychoses. Very few studies on gene expression of antipsychotic-naive bipolar disorder patients have been conducted,^15, 16, 17, 18^ reporting alterations in inflammatory genes, such as *TNF*,^15^ and in genes of AKT1/mTOR pathway.^18^

Our objectives are to investigate differences in the messenger RNA (mRNA) levels of 12 genes among individuals with FEP of schizophrenia-spectrum disorder (SCZ), FEP with mania (BD) and healthy controls. We also want to verify whether gene expression is correlated to clinical features, including functional impairment and severity of psychotic, manic, and depressive symptoms. Particularly, we compared SCZ with BD to identify diagnostic specificity (genes related to manic symptoms), and FEP (both SCZ and BD) and healthy controls to find genes related to psychosis itself as a broad syndrome.

To our knowledge, this is the first study that compares gene expression between antipsychotic-naive FEP of schizophrenia spectrum disorder and FEP with mania. The study aims are to differentiate BD and SCZ, improving early diagnosis and adequate intervention.

We selected the genes based on their biological role in psychotic disorders. As a second criterion, these genes should be expressed in whole blood (according to information available in http://www.genecards.org). We selected genes related to dopamine neurotransmission (*COMT*), inflammation and the immune system (*TNF*), neurodevelopment (*DISC1*, *PAFAH1B1* and *NDEL1*), myelination (*MBP*), cell signaling (*AKT1*), the microRNA machinery (*DGCR8*, *DICER1* and *DROSHA*), protein degradation (*UFD1L*) and adhesion (*DGCR2*). Some genes were selected mainly because of their location in the 22q11.2 region (*COMT*, *DGCR2*, *DGCR8* and *UFD1L*), as its deletion is one of the strongest known genetic risk factors for psychotic disorders.^19^ We previously analyzed the same genes in other studies comparing FEP (excluding individuals with bipolar disorder diagnosis) with ultra-high risk individuals and controls, with positive findings.^11, 12, 20, 21, 22, 23^ Here a comparison between FEP of schizophrenia spectrum disorder (SCZ) and FEP with mania (BD) is presented, as well as between BD and healthy controls.

## Materials and methods

### Study population

The Research Ethics Committee of UNIFESP approved the research protocol, and all participants and family members provided written informed consent prior to enrollment in the study (CEP 0603/10).

Antipsychotic-naive FEP patients (*N* =69) were recruited from a psychiatric emergency unit in São Paulo, Brazil. The diagnosis of a psychotic disorder was established by trained psychiatrists based on the criteria of the Diagnostic and Statistical Manual of Mental Disorders, Fourth Edition (DSM-IV), using the Structured Clinical Interview of the DSM-IV. Patients were assessed at baseline and followed up for at least 2 months. At the end of follow-up, patients with a schizophrenia or schizophreniform disorder diagnosis were classified as FEP of schizophrenia spectrum disorder (SCZ, *N* =53) and patients who met bipolar disorder (with psychotic symptoms) diagnostic criteria were classified as FEP with mania (BD, *N* =16).

Inclusion criteria were age between 16 and 40 years and no previous history of antipsychotic medication. Prior treatment with benzodiazepines was allowed. Patients with psychotic episodes due to a general medical condition, substance-induced psychotic disorder, intellectual disability or psychotic episodes that were associated with major depressive disorder were excluded.

The patients were assessed by: (a) PANSS (Positive and Negative Syndrome Scale);^24^ (b) CGI (Clinical Global Impression Scale);^25^ (c) GAF (Global Assessment of Functioning Scale), (d) CDSS (Calgary Depression Scale for Schizophrenia);^26^ and (e) YMRS (Young Mania Rating Scale). PANSS dimensions were derived from a previous study in a Brazilian population.^27^

The healthy control group (*N* =73) comprised age- and gender-matched volunteers with no current or previous psychiatric diagnoses or first-degree family history of psychotic disorders.

Peripheral blood samples were collected prior to the administration of antipsychotics (for patients) or after psychiatric interview (for controls).

### Analysis of transcript levels of selected genes

A total of 5 ml of whole blood was collected in PAXgene RNA tubes (PreAnalytix, Hombrechtikon, Switzerland). RNA was isolated using a PAXgene Blood RNA kit (Qiagen, Germantown, MD, USA) according to the manufacturer's instructions. RNA integrity was determined through electrophoresis on a 1.0% agarose gel, and the quality and quantity of the RNA samples were determined using a NanoDrop ND-1000 spectrophotometer (Nanodrop, Wilmington, DE, USA).

Approximately 400 ng of each RNA sample was reverse-transcribed using a High-Capacity cDNA Reverse Transcription Kit (Life Technologies, Foster City, CA, USA). Then, 20 to 100 ng of complementary DNA (cDNA) was diluted in H_2_O, mixed with TaqMan Universal PCR Master Mix (Life Technologies), and loaded on Taqman Low-Density Array (TLDA) microfluidic cards (Life Technologies). Probes and primers of 12 target genes, two housekeeping genes (*ACTB* and *GAPDH*), and one positive control for the reaction (*18S*) were preloaded in the 384 wells (in triplicates) of each TLDA card (Life Technologies). Assays and the exons and transcripts that they recognize are described in Supplementary Table 1. The experiments were performed in accordance with the manufacturer's instructions using the ViiA 7 Real-Time PCR System (Life Technologies).

Genes were selected based on their previously reported association with psychotic disorders and their expression in blood (http://www.genecards.org/).

Gene expression was quantified using the relative threshold method (Crt) with the geometric mean (GM) between *ACTB* and *GAPDH* as the endogenous control. Delta cycle relative threshold values (ΔCrt=Crt_target gene_−Crt_GM_) were calculated for each sample and 2^-ΔCrt^ values were included in the PASW Statistics (version 18.0, SPSS, Chicago, IL, USA) data set.

### Statistical analysis

Sample size was chosen using G*Power 3.1.6 software (Heinrich-Heine-Universität Düsseldorf, Düsseldorf, Germany) and considering an analysis of variance test, with medium effect size *f*=0.30, *α*=0.05; power=0.80 and 3 groups. Two-sided tests were used for statistical analyses. For continuous variables, normality and homogeneity was checked with Shapiro–Wilk and Levene tests and log-transformed as needed. Gender and age differences between the SCZ, BD and control group were verified using the *χ*^2^-test and analysis of variance test, respectively. Differences in severity of symptoms and functional impairment (CDSS, CGI, GAF and YMRS total scores and PANSS scores) were compared using Student's *t*-test.

2^-ΔCrt^ values were compared among SCZ, BD and control groups using general linear model (GLM), with gender as a fixed factor if necessary and with Bonferroni correction for multiple comparisons (12, considering the number of genes assessed). *Post hoc* comparisons were carried out using the Bonferroni test. In addition, for genes that were differentially expressed, we verified the correlation between the 2^-ΔCrt^ values and the clinical aspects using linear regression and inserting group (SCZ or BD) as an independent variable. In order to evaluate the predictive power of these genes, receiver-operating characteristic curves were obtained, and the respective statistical significance were assessed by *χ*^2^-test on the area under the curve (AUC). The significance level was set at 5%.

## Results

The clinical and demographic characteristics of the participants are presented in Table 1.

SCZ patients exhibited higher scores on the negative dimension of the PANSS, and lower scores on the excitement compound of the PANSS and YMRS, than BD patients (Table 1). In addition, BD patients presented with poorer global function, as indicated by GAF (Table 1), compared with SCZ patients. No significant age or gender differences were observed among the different groups.

### Analysis of transcript levels of selected genes among antipsychotic-naive SCZ, BD patients and healthy controls

Gene expression results comparing the three groups (SCZ, BD and healthy controls) are reported in Table 2. Four genes were found to be differentially expressed among the groups after Bonferroni correction for 12 comparisons. Of them, two genes (*MBP* and *NDEL1*) exhibited higher expression levels in antipsychotic-naive patients (both SCZ and BD) than in healthy controls (Table 2). Moreover, the expression of two other genes, *AKT1* and *DICER*, was higher in BD than in both SCZ patients and controls (Table 2).

The expression of these four genes was not correlated with prior benzodiazepine administration (*P*>0.05) or age (*P*>0.05).

We analyzed the receiver-operating characteristic curve and we found the same results: *AKT1* and *DICER1* 2^-▵Crt^ values differentiated BD from SCZ patients (*AKT1* AUC=0.768, *P*=0.001; *DICER1* AUC=0.812, *P*=1.706 × 10^-4^; Supplementary Figure 1) and healthy controls (*AKT1* AUC=0.798, *P*=2.129 × 10^-4^; *DICER1* AUC=0.865, *P*=5.525 × 10^-6^; Supplementary Figure 2), but not SCZ from healthy controls (*AKT1* AUC=0.505, *P*=0.927; *DICER1* AUC=0.561, *P*=0.246; Supplementary Figure 3). On the other hand, *MBP* and *NDEL1* ▵Crt values did not distinguish SCZ from BD (*MBP* AUC=0.597, *P*=0.267; *NDEL1* AUC=0.553, *P*=0.525; Supplementary Figure 1), but both could differentiate SCZ (*MBP* AUC=0.677, *P*=0.006; *NDEL1* AUC=0.660, *P*=0.002; Supplementary Figure 3) and BD patients (*MBP* AUC=0.805, *P*=2.856 × 10^-4^; *NDEL1* AUC=0.728, *P*=0.004; Supplementary Figure 2) from healthy controls.

### Correlation between gene expression and clinical characteristics

We tested the correlation of differential gene expression (*AKT1*, *DICER*, *MBP* and *NDEL1*) and the severity of symptoms and functional impairment (CDSS, CGI, GAF and YMRS total scores, and PANSS scores) using linear regression and inserting group as an independent variable. However, no significant correlation was detected after Bonferroni correction for multiple comparisons.

## Discussion

In the present study, we identified gene expression alterations that might be linked to manic and psychotic features. We primarily detected that *AKT1* and *DICER1* expression levels were higher in BD patients compared with SCZ patients and controls, suggesting that the expression of these genes is associated more specifically to manic features. In addition, *MBP* and *NDEL1* expression levels were higher in both SCZ and BD patients than in healthy controls, indicating that these genes may be related to psychosis *per se* (independently of diagnosis). We described similar results comparing FEP (excluding BD) and controls in a previous study.^22^ Here we focused on verifying if these changes in gene expression are also observed in BD, and moreover, if they can discriminate BD from SCZ patients. Although all these four genes have a role in central nervous system, this study aimed to find potential biomarkers, even if they might not be related to the pathophysiology of the disease. A brief description of each comparison (SCZ × BD and BD × control) and the relationship of each gene with psychotic disorders are described below.

### SCZ and BD

Two genes were differentially expressed in BD, when compared with both SCZ and healthy controls, namely *AKT1* (V-Akt Murine Thymoma Viral Oncogene Homolog 1) and *DICER1* (Dicer 1, ribonuclease type III).

*AKT1*, which encodes a serine–threonine protein kinase, is involved in a variety of central nervous system functions such as neurodevelopment, synaptic plasticity and protein synthesis.^28^ Moreover, AKT1 was shown to facilitate dopamine signaling,^29, 30^ and to regulate a wide array of cellular processes, such as metabolism, growth, proliferation and apoptosis.^31^ AKT1 participates in the PI3K/Akt/mTOR pathway, which is important for many immunological mechanisms.^32^

In addition, lithium, antidepressants, antipsychotics and other mood stabilizers seem to increase phosphorylation of AKT.^33, 34, 35^ A decrease in AKT1 protein and mRNA levels was found in postmortem brain tissue and lymphocyte-derived cells of individuals with schizophrenia and bipolar disorder, compared with healthy controls.^35, 36, 37^ Although these studies primarily suggest an AKT1 deficiency, a recent study^14^ found increased *AKT1* expression levels in peripheral blood of antipsychotic-naive schizophrenia patients. Kumarasinghe *et al.* (2013) also observed that antipsychotic pharmacotherapy could partially compensate for this upregulation, providing further evidence for a link between AKT1 and dopaminergic transmission.

Although we did not find differences in *AKT1* mRNA levels between SCZ and controls, we found that this gene was upregulated specifically in BD patients. A previous study in a Brazilian sample of unmedicated, depressed individuals with bipolar disorder showed decreased *AKT1* expression in the blood.^18^ Our finding of increased *AKT1* expression in patients on the opposite pole of the spectrum (in mania) may suggest an association between *AKT1* expression and mood pole.

*DICER1* synthesizes DICER, a member of the ribonuclease III protein family that is involved in the generation of microRNAs (miRNAs), which regulate gene expression at the posttranscriptional level.^38^ MiRNAs are 22-nt-long RNAs generated from longer precursor RNAs. In general, they repress translation, but they can also acquire other functions after binding to their target RNA. Notably, many studies implicated miRNAs in the development of psychotic disorders.^39, 40^

DICER has an important role in the development and function of the immune^41^ and central nervous systems.^42^ *DICER1* is upregulated in the dorsolateral prefrontal cortex^42, 43^ and lymphoblastoid cell lines^44^ of schizophrenia cases. In addition, *DICER1* single-nucleotide polymorphisms^45^ and copy-number variations^46^ are associated to schizophrenia. Notably, valproic acid, a mood stabilizer used to treat bipolar disorder, induces DICER degradation.^47^

### BD and healthy controls

Four genes were upregulated in BD patients compared with healthy controls (*AKT1*, *DICER1*, *MBP* and *NDEL1*). *AKT1* and *DICER1* expression levels were different between BD and SCZ patients, whereas the expression levels of *MBP* (myelin basic protein) and *NDEL1* (nuclear distribution of protein nudE-like 1) were similar. Notably, in our previous findings in a larger cohort of FEP without bipolar disorder patients we found that *MBP* and *NDEL1* were upregulated in antipsychotic-naive patients compared with controls.^22^ Here we show that these genes are also dysregulated in FEP with mania.

Myelin-related pathways are involved in the aetiologies of both schizophrenia and bipolar disorder.^48^ The *MBP* gene produces two families of structurally related proteins from different promoters: the MBP and the Golli (gene in the oligodendrocyte lineage) isoforms. In our study, we used an assay that can detect both types of isoforms (one Golli—NM_001025101, and two classic MBP isoforms—NM_001025090 and NM_001025092; Supplementary Table 1). Owing to the higher expression of Golli isoforms in the immune system,^49^ we may assume that they represent the expression detected in our experiments. The biological function of Golli isoforms involves myelin formation and maintenance,^50^ oligodendrocyte proliferation and migration,^51^ and calcium homeostasis.^52^ This specific calcium pathway is altered in schizophrenia and bipolar disorder^53^ and is also affected by antipsychotic medications.^54^

*MBP* expression studies in postmortem tissues from different brain regions revealed an association with psychotic disorders, suggesting that MBP mRNA and protein levels (of the classic isoforms) are decreased in patients with schizophrenia ^55, 56, 57^ or bipolar disorder.^58^ However, no differences were found in the Golli-MBP mRNA levels in postmortem dorsolateral prefrontal cortex samples of schizophrenia patients compared with controls.^59^ Notably, *MBP* expression is affected by antipsychotic treatment,^60, 61^ and hence, analyzing antipsychotic-naive patients is essential to attenuate the effects of these medications on gene expression.

In our study, both antipsychotic-naive SCZ and BD patients exhibited increased *MBP* expression levels (most likely Golli isoforms) compared with healthy controls, suggesting that this gene might be associated to psychosis *per se*, as both patient groups exhibited psychotic symptoms. Indeed, when we analyzed FEP patients in a larger sample, excluding those with manic features, *MBP* was also upregulated compared with healthy controls^22^ and to ultra-high risk individuals,^23^ supporting that MBP might have a role in psychosis. Moreover, in an independent report by Kumarasinghe *et al.* (2013), antipsychotic-naive schizophrenia patients exhibited higher *MBP* mRNA levels, compared with controls. After 6 to 8 weeks of risperidone or haloperidol treatment, the *MBP* mRNA levels were similar to that of controls.^14^ However, another study did not reveal significant changes in *MBP* in the peripheral blood of first-episode schizophrenia and bipolar disorder patients.^17^

NDEL1 is encoded by a gene located at chromosome 17p13.1, and is robustly expressed in developing and mature neurons in the brain. It has been suggested to have a role in neuronal migration during embryogenesis.^62^ Other well-known functions of NDEL1 include cytoskeleton organization, cell proliferation and survival regulation, oligopeptidase activity (potential neuropeptidase activity), neuritogenesis, neuronal migration and cell signaling.^63, 64, 65^ This protein was first discovered due to its enzyme activity on neuropeptides,^66^ and second because of its ability to form complexes with other proteins such as LIS1, which is encoded by the *PAFAH1B1* (platelet-activating factor acetylhydrolase 1b, regulatory Subunit 1 (45 kDa)) gene that is mutated in lissencephaly, a rare brain formation disorder.^67, 68^ In addition, NDEL1 is able to bind DISC1,^65^ a psychosis-associated protein that is the product of a well-known schizophrenia risk factor gene,^69^ and is associated with bipolar disorder.^70^

Gene expression studies conducted in both brain and peripheral tissues revealed reduced *NDEL1* expression levels in schizophrenia patients.^71, 72^ However, none of these studies have investigated antipsychotic-naive subjects or patients at the first stages of psychotic disorders. In a similar analysis, we have previously reported an upregulation of *NDEL1* gene expression in FEP patients compared with healthy controls.^22^As we have included BD patients in the present study, we suggest that *NDEL1* expression is also altered in psychotic disorders with manic features, in the same way as observed in SCZ patients. Moreover, a subgroup of FEP patients with depression showed lower levels of *NDEL1* expression,^20^ opposite to our finding in BD, which is also at the opposite pole of depression. Thus, higher *NDEL1* expression might be characteristic of BD and FEP without depression, while lower *NDEL1* expression levels could be associated to FEP with depression.

The results of this study need to be replicated in additional studies and should be interpreted in light of some limitations. First, the sample size of the groups and particularly the BD patient group was small, and, hence, it may lack power to identify some gene expression differences. Second, it was a cross-sectional study, and the follow-up of these patients would confirm the diagnosis and may help to find markers for response to treatment. Third, we did not have a group of patients with mania without psychotic symptoms. Such a group would help to define which genes are specifically related to mania. Fourth, we cannot assure if our findings in whole blood translate to what occurs in the brain. Considering that whole blood presents a mixture of various leukocyte subtypes, our gene expression findings may also be partially confounded by various proportions of leukocyte subtypes.^73^ However, an important strength is our focus on unmedicated patients in the first stages of the disorder. Consequently, we needed to use a biological material that can be easily collected, though we acknowledge that peripheral markers may not necessarily reflect brain pathophysiology.

## Conclusions

To our knowledge, this is the first study that compares gene expression in antipsychotic-naive FEP of SCZ and FEP with mania (BD), suggesting potential diagnostic specificities. On the basis of an integrated model, we propose that *MBP* and *NDEL1* are upregulated in SCZ and BD patients, who all exhibit psychotic symptoms. Moreover, two other genes, *AKT1* and *DICER1*, were upregulated in BD patients only, indicating that these genes could be related to mania, independently of psychotic symptoms. Although further validation in a large sample is still needed, our findings suggest that genes related to neuronal development are altered in psychotic disorders, and some of them might support the differential diagnosis between schizophrenia and bipolar disorder in the near future, which in turn could have an impact on the treatment of these disorders.

## Figures and Tables

**Table 1:** Clinical and demographic characteristics of the participants

*Variable*	*Controls*	*SCZ*	*BD*	*Test value; df;* P*-value*
*Gender*				
Males (%)	42 (57.5)	34 (64.2)	12 (75)	
Females (%)	31 (42.5)	19 (35.8)	4 (25)	*χ*^2^=1.869; df=2; *P*=0.393
				
Age in years; mean (s.d.)	25.66 (7.31)	26.34 (7.53)	25.13 (6.98)	F(2,139)=0.220; *P*=0.803
PANSS negative; mean (s.d.)		27.32 (10.38)	15.67 (6.80)	*t*=3.692; df=60; *P*= 4.826 × 10^-4^*
PANSS disorganization/cognition; mean (s.d.)		27.56 (7.91)	29.09 (6.73)	*t*=−0.640; df=59; *P*=0.524
PANSS excitement; mean (s.d.)		26.42 (10.39)	37.68 (11.17)	*t*=−2.946; df=60; *P*=0.005*
PANSS positive; mean (s.d.)		35.94 (6.97)	35.18 (7.97)	*t*=0.433; df=59; *P*=0.666
PANSS depression/anxiety; mean (s.d.)		25.33 (9.29)	24.83 (7.23)	*t*=−0.066; df=59; *P*=0.948
PANSS total; mean (s.d.)		96.27 (20.09)	91.55 (19.21)	*t*=0.709; df=58; *P*=0.481
GAF; mean (s.d.)		31.96 (8.81)	20.43 (9.60)	*t*=3.711; df=16.957; *P*=0.002*
CGI; mean (s.d.)		4.87 (0.74)	5.36 (1.15)	*t*=−1.183; df=16.062; *P*=0.254
CDSS; mean (s.d.)		4.85 (5.32)	1.00 (1.49)	*t*=1.416; df=36; *P*=0.165
YMRS; mean (s.d.)		11.63 (10.87)	27.23 (12.12)	*t*=−4.242; df=36.088; *P*=1.473 × 10^-4^*

Abbreviations: BD, first-episode of psychosis—mania with psychosis; CDSS, Calgary Depression Scale for Schizophrenia; CGI, Clinical Global Impression Scale; df, degrees of freedom; GAF, Global Assessment of Functioning Scale; PANSS, Positive and Negative Syndrome Scale; SCZ, first-episode of psychosis—schizophrenia spectrum; YMRS, Young Mania Rating Scale.

*P<0.05.

**Table 2:** 2^−ΔCrt^ values of the 12 genes in the antipsychotic-naive SCZ and BD patients and controls

*Post hoc* P*-value*							
*Gene*	*SCZ 2*^−ΔCrt^; *mean (*s.d.*)*	*BD 2*^−ΔCrt^; *mean (*s.d.*)*	*HC 2*^−ΔCrt^; *mean (*s.d.*)*	*Test value; adjusted* P*-value*	*Post hoc p-value SCZ vs BD*	*Post hoc p-value* *BD vs HC*	*Post hoc p-value* *SCZ vs HC*
*AKT1*	0.053 (0.011)	0.057 (0.009)	0.050 (0.007)	F(2,138)=8.088; *P*=0.006^a^	0.001^a^	4.86 × 10^-4^^a^	1.000
*COMT*	0.020 (0.003)	0.022 (0.003)	0.021 (0.004)	F(2,134)=2.018; *P*=1.000	0.186	0.896	0.520
*DGCR2*	0.042 (0.009)	0.043 (0.008)	0.045 (0.006)	F(2,135)=0.524; *P*=1.000	0.836	0.499	1.000
*DGCR8*	0.008 (0.002)	0.008 (0.002)	0.008 (0.002)	F(2,133)=3.214; *P*=0.520	0.150	1.000	0.084
*DICER*	0.024 (0.007)	0.029 (0.007)	0.021 (0.005)	F(2,137)=14.979; *P*=1.57 x10^-5^^a^	6.66 × 10^-5^^a^	6.14 × 10^-7^^a^	0.483
*DISC1*	0.003 (0.002)	0.004 (0.002)	0.003 (0.001)	F(2,98)=2.649; *P*=0.910	0.111	1.000	0.245
*DROSHA*	0.010 (0.003)	0.010 (0.003)	0.012 (0.004)	F(2,123)=1.228; *P*=1.000^b^	0.508	1.000	0.150
*MBP*	0.003 (0.002)	0.004 (0.001)	0.002 (0.001)	F(2,97)=8.729; *P*=0.004^a^	1.000	0.002^a^	0.004^a^
*NDEL1*	0.073 (0.018)	0.076 (0.014)	0.060 (0.012)	F(2,138)=6.555; *P*=0.023^a^	1.000	0.030^a^	0.006^a^
*PAFAH1B1*	0.040 (0.008)	0.040 (0.006)	0.038 (0.006)	F(2,135)=1.246; *P*=1.000	0.352	0.559	1.000
*TNF*	0.008 (0.003)	0.006 (0.002)	0.008 (0.002)	F(2,126)=0.384; *P*=1.000^b^	0.507	0.405	1.000
*UFD1L*	0.006 (0.002)	0.006 (0.001)	0.006 (0.002)	F(2,135)=0.406; *P*=1.000	1.000	1.000	1.000

Abbreviations: *AKT1*, V-Akt murine thymoma viral oncogene homolog 1; BD, first-episode of psychosis—mania with psychosis; *COMT*, catechol-*O*-methyltransferase; HC, healthy controls; *DGCR2*, Digeorge syndrome critical region gene 2; *DGCR8*, Digeorge syndrome critical region gene 8; *DICER1*, Dicer 1, ribonuclease type III; *DISC1*, disrupted-in-schizophrenia 1; *DROSHA*, Drosha, ribonuclease type III; *MBP*, myelin basic protein; *NDEL1*, nuclear distribution protein nudE-like 1; *PAFAH1B1*, platelet-activating factor acetylhydrolase 1b, regulatory subunit 1 (45 kDa) or LIS1; SCZ, first-episode of psychosis—schizophrenia spectrum; *TNF*, tumor necrosis factor; *UFD1L*, ubiquitin fusion degradation 1 like (yeast).

a Significant *P*-values.

b Gender was inserted as a fixed factor. Adjusted *P*-values were obtained after Bonferroni correction for multiple comparisons (12 comparisons).
