# Supplementary material for: Gene expression alterations related to mania and psychosis in peripheral blood of patients with a first episode of psychosis
Source: Transl Psychiatry. 2016 Oct 4;6(10):e908–. doi: 10.1038/tp.2016.159 (PMC5315542; doi:10.1038/tp.2016.159)
Supplement: Supplementary Information [file tp2016159x1.doc]

**Supplementary Table 1:** Assays used in the real time PCR, and the transcripts and their exons that they recognize.

| **Gene symbol** | **Assay ID** | **Refseq mRNA** | **Exon boundary** |
| --- | --- | --- | --- |
| *AKT1* | Hs00178289_m1 | NM_001014431.1  NM_001014432.1  NM_005163.2 | 4 – 5  5 – 6  4 – 5 |
| *COMT* | Hs02511558_s1 | NM_000754.3  NM_001135161.1  NM_001135162.1  NM_007310.2 | 6 – 6  6 – 6  6 – 6  4 – 4 |
| *DGCR2* | Hs00192970_m1 | NM_001184781.1  NM_005137.2 | 1 – 2  1 – 2 |
| *DGCR8* | Hs00256062_m1 | NM_001190326.1  NM_022720.6 | 3 – 4  3 – 4 |
| *DICER1* | Hs00229023_m1 | NM_001195573.1  NM_001271282.1  NM_030621.3  NM_177438.2 | 13 – 14  14 – 15  15 – 16  14 – 15 |
| *DISC1* | Hs00257791_s1 | AK023443.1 | 1 – 1 |
| *DROSHA* | Hs00203008_m1 | NM_001100412.1  NM_013235.4 | 26 – 27  26 – 27 |
| *MBP* | Hs00922788_m1 | NM_001025090.1  NM_001025092.1  NM_001025101.1 | 1 – 2  1 – 2  4 – 5 |
| *NDEL1* | Hs00229366_m1 | NM_030808.4 | 8 – 9 |
| *PAFAH1B1* | Hs00181182_m1 | NM_000430.3 | 6 – 7 |
| *TNF* | Hs01113624_g1 | NM_000594.3 | 2 – 3 |
| *UFD1L* | Hs00799945_s1 | NM_001035247.2  NM_005659.6 | 6 – 6  6 – 6 |


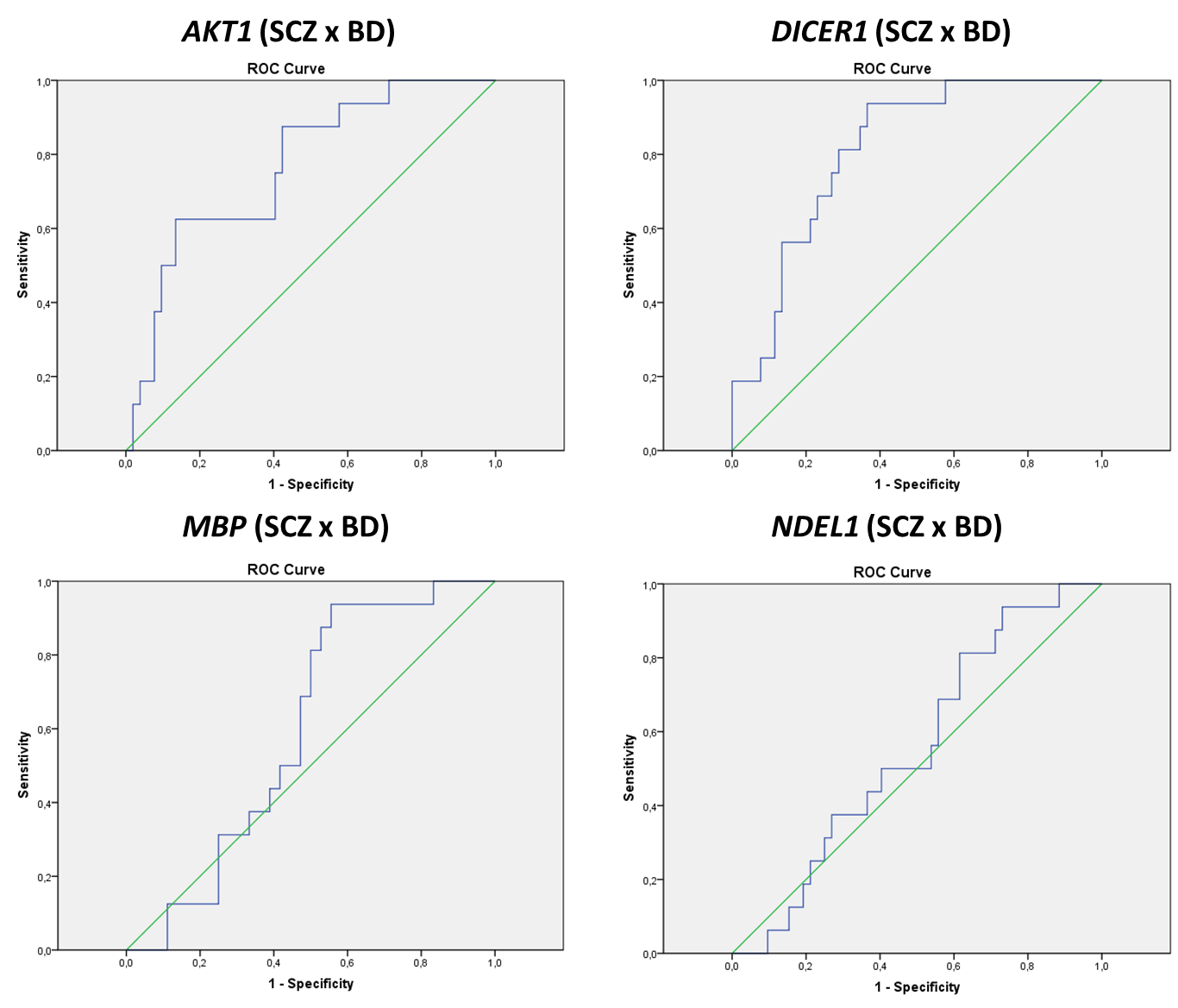


**Supplementary Figure 1:** Receiver operating characteristic (ROC) curves comparing *AKT1*, *DICER1*, *MBP*, and *NDEL1* 2^-∆Crt^ values between SCZ (First-episode of psychosis-Schizophrenia Spectrum) and BD (Fist-episode of psychosis-Mania with psychosis), considering BD as the positive group.


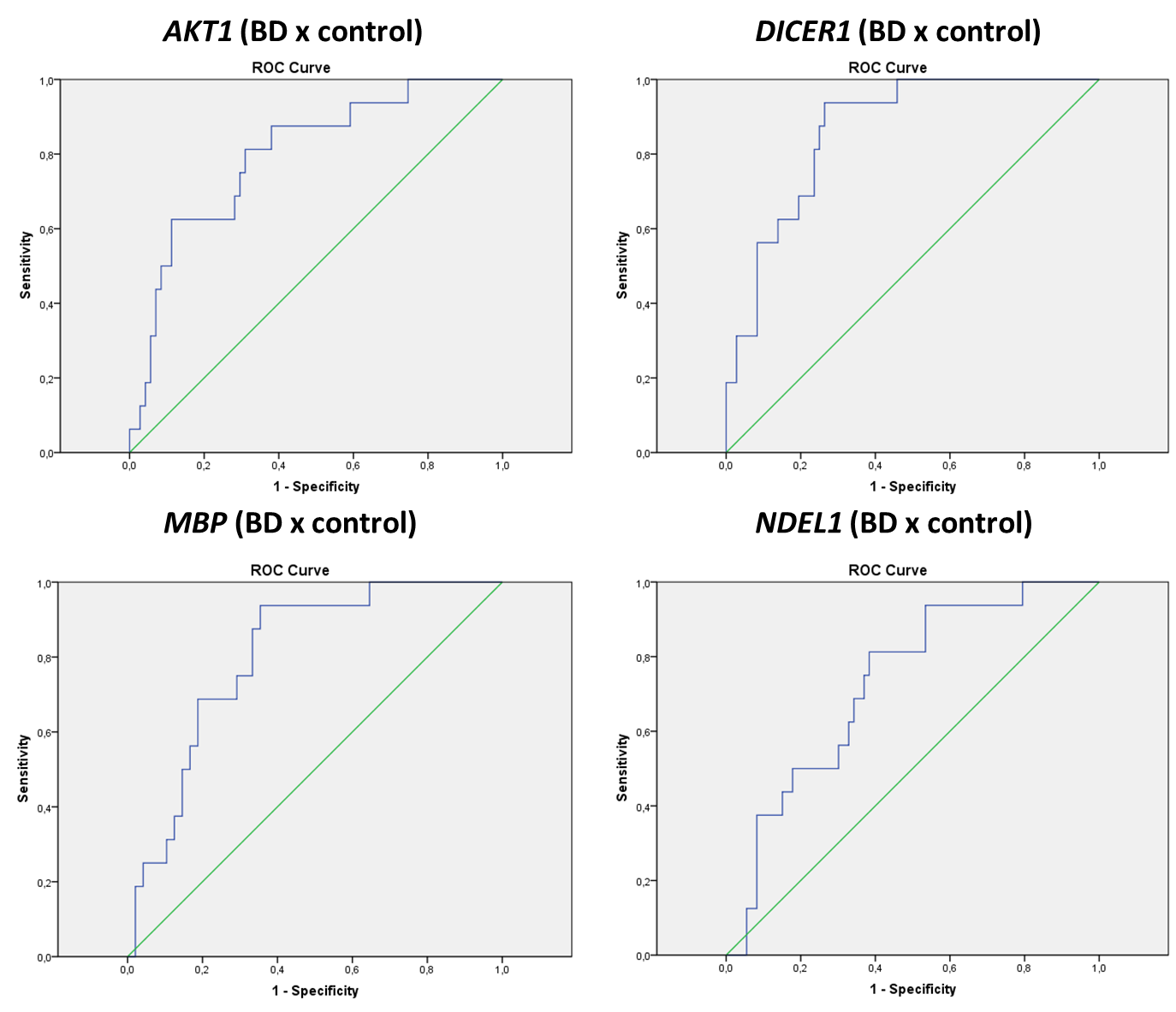


**Supplementary Figure 2:** Receiver operating characteristic (ROC) curves comparing *AKT1*, *DICER1*, *MBP*, and *NDEL1* 2^-∆Crt^ values between BD (Fist-episode of psychosis-Mania with psychosis) and controls, considering BD as the positive group


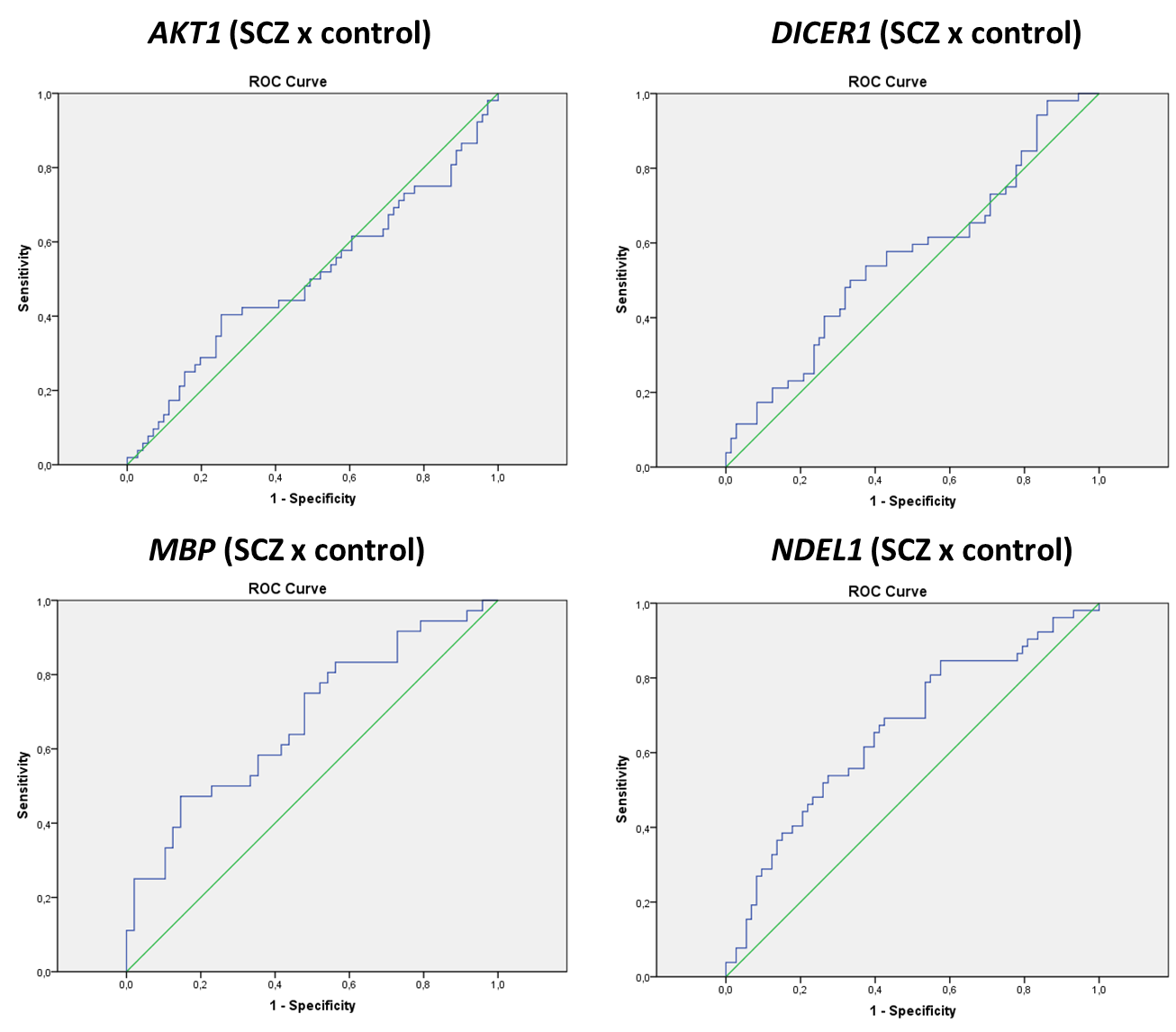


**Supplementary Figure 3:** Receiver operating characteristic (ROC) curves comparing *AKT1*, *DICER1*, *MBP*, and *NDEL1* 2^-∆Crt^ values between between SCZ (First-episode of psychosis-Schizophrenia Spectrum) and controls, considering SCZ as the positive group.
